# Evaluation of Antibacterial Activity of Madhuca longifolia (Mahua) Stem Extract Against Streptococcus mutans: An In Vitro Study

**DOI:** 10.7759/cureus.52210

**Published:** 2024-01-13

**Authors:** Areej Fatma, Vipin Ahuja, Annapurna Ahuja, Satish Kumar, Sunit K Srivastava, Nilima R Thosar

**Affiliations:** 1 Pediatric and Preventive Dentistry, Hazaribag College of Dental Sciences and Hospital, Hazaribagh, IND; 2 Pediatric and Preventive Dentistry, Government Dental College and Hospital, Jamnagar, IND; 3 Periodontics and Implant Dentistry, Hazaribag College of Dental Sciences and Hospital, Hazaribagh, IND; 4 University Department of Biotechnology, Vinoba Bhave University, Hazaribagh, IND; 5 Pediatric and Preventive Dentistry, Sharad Pawar Dental College and Hospital, Datta Meghe Institute of Medical Sciences, Wardha, IND

**Keywords:** chewing stick, madhuca longifolia, chlorhexidine, madkam kaarkad, traditional plants, dental caries

## Abstract

Introduction

*Madhuca longifolia* is one of the important folklore medicinal plants with a plethora of established pharmaceutical properties. Its twigs are used as chewing sticks (toothbrushes), and it is believed that if a person uses it daily, it will make their gum healthy and strong. No study has ever been conducted to evaluate the antibacterial effect of *M. longifolia* extracts against oral microorganisms.

Materials and methods

Fresh stem twigs (Madkam Kaarkad) of *M. longifolia* were collected and dried. The dried stem was cut into small pieces, 5 g of which was mixed with 50 ml distilled water (in the ratio 1:10) and kept for two days for maceration. After two days, the liquid was filtered and the final filtrate was obtained, from which dry pellets were made and stored in the refrigerator at 4°C. Brain heart infusion agar was used as a medium to grow the lyophilized bacteria. Pure strains of *Streptococcus mutans* 890 were obtained from the Microbial Type Culture Collection (MTCC) and MTCC-suggested protocol was followed for the revival of lyophilized bacteria. The agar well diffusion method was used to determine the zone of inhibition. The extract of stems with different concentrations (10%, 7.5%, 5.0%, and 2.5%) and at different volumes (100 µl, 150 µl, 200 µl, and 250 µl) was transferred to the agar plates. Chlorhexidine 0.2% was used as a control and it was also transferred to agar plates, which were incubated aerobically at 37°C for 24 hours. Antibacterial activity was interpreted from the size of the diameter of zones of inhibition measured in millimeters using a measuring scale in all the agar plates.

Results

The minimum zone of inhibition of 11 mm at 2.5% concentration and 100 µl volume of *M. longifolia* extract and the maximum zone of inhibition of 20 mm at 10% concentration and 250 µl volume was notified. While for chlorhexidine at 0.2% concentration, the zone of inhibition obtained was 9.5 mm at 40 µl volume. The minimum inhibitory concentration (MIC) value of *M. longifolia *was found to be 35 mg/ml.

Conclusion

*M. longifolia *showed marked antibacterial activity against *S. mutans *and has a high MIC value. Therefore, this plant can be considered an effective agent against oral diseases like dental caries.

## Introduction

Indisputable evidence proves *Streptococcus mutans* is the headman microorganism accountable for dental caries. The accountability of this microorganism for its cariogenic properties is concealed in three fundamental elements: first is its skill to produce enormous extracellular polymers of glucan from sucrose, which to a great extent augments extracellular polymeric matrix formation; second is its acidogenicity, where carbohydrates metabolize to acids; and third is aciduricity, which gives it survival in low pH conditions. Several synthetic medicines are useful in the preventive arena of dentistry, with wide harmful possessions. One of the sizable global issues faced today is antibiotic resistance. Acquired antibiotic resistance occurs when bacteria formerly sensitive to antibiotics develop confrontation to the same medicine, which is now feared as an alarming pandemic delinquency due to lenient misuse of synthetic medicine in the health industry. This misdemeanor prevails more in developing countries where the ratio of the well-qualified health workforce to the population is at scarcity and quackery practices with ill-logical use of medicines are at flow [1,2].

Therefore, herbal cures are now preferred, as it is effective and affordable. In Indian culture, plants as medicinally effective media have been used for centuries to mend oral health and hygiene positively. History also documents the use of plant twigs and branches as toothbrushes by Indian tribes. Rural people utilize a wide variety of plant species as toothbrushes. One of them is the twig of the mahua tree, which is popularly known as Mahuadatun or Madkam Kaarkad by the Ho tribes of Jharkhand (India). The scientific name of this plant is *Madhuca longifolia* (J.Konig) J.F.Macbr of the family *Sapotaceae* [3-5]. This plant was chosen for the present study because there is a general belief that if a person uses it daily, it will make their gum healthy and strong and it prevents teeth from falling even among aged people. There is a saying among tribes and native people that “Chew the Madkam Kaarkad every day and you can chew even stones.” *Madhuca longifolia* is antimicrobial and effective against *Bacillus subtilis*, *Pseudomonas aeruginosa*, *Escherichia coli*, and many others [6,7]. Still, there is no documented research on *M. longifolia* extracts testing its antimicrobial efficacy on oral pathogens. Therefore, the study was undertaken to gauge the in vitro antibacterial activity of *M. longifolia* against *S. mutans*.

## Materials and methods

Twigs (Madkam Kaarkad) of the *M. longifolia* plant were collected from the outskirts of Hazaribagh, a district town of Jharkhand, and were authenticated by an expert from the Plant Collection Centre, Institute of Forest Productivity, Lalgutwa, Ranchi, Jharkhand, India. Pure strains of *S. mutans* 890 were acquired from the Microbial Type Culture Collection (MTCC), Institute of Microbial Technology, Chandigarh, India. Brain heart infusion (BHI) broth was obtained from ReadyMED, Chaitanya Agro Biotech Pvt. Ltd., Malkapur, Maharashtra.

Preparation of *Madhuca longifolia* extract

After the collection of fresh stems twigs of *Madhuca longifolia*, the twigs were prepared by washing with tap water and double distilled water thrice and twice, respectively. It was allowed to dry in the shade for seven days and then in a hot air oven for one day at a temperature of 40°C. To prepare plant extract, the maceration technique for the extraction of medicinal and aromatic plants (MAPs) as proposed by Singh [8] was used to prepare stock solution. The dried stem was cut into small pieces, 5 g of which were admixed to 50 ml distilled water (ratio 1:10) and kept for two days for maceration. After two days, the liquid was filtered first by using a muslin cloth and again filtered by using Whatman filter paper to obtain the last filtrate from which dry pellets weighing 462 mg were obtained by evaporation of solvent at 40°C in a hot air oven. Stock solution 10% was prepared by adding 462 mg of dry pellets in 4.62 ml of distilled water. It was kept in the refrigerator at 4°C for further investigations. Various concentrations were obtained from this absolute stock.

Preparation of medium and bacterial culture

The plating medium used to cultivate freeze-dried bacteria was BHI agar. The MTCC-suggested protocol was followed for the revival of lyophilized bacteria. The circular disks with known different concentrations of antibacterial agent were placed in agar plates and the zones of inhibition were evaluated with the agar well diffusion procedure [9]. The stem extracts with different concentrations (10%, 7.5%, 5.0%, and 2.5%) and at different volumes (100 µl, 150 µl, 200 µl, and 250 µl) were transferred to the agar plates. The control taken to compare antibacterial activity was 0.2% chlorhexidine and was incubated at 37°C for 24 hours on agar plates aerobically. The size (diameter) of zones of inhibition was calculated in millimeters (mm) with a measuring scale and correspondingly antibacterial activity was equated.

Minimum inhibitory concentration

Minimum inhibitory concentration (MIC) was determined using the previously described macrodilution procedure with some modification in the BHI broth medium [10,11]. In brief, 500 µl of bacterial culture containing 1 x 106 CFU/ml bacteria was added separately to eight different tubes (T_od_) having 400 µl BHI broth and 100 µl of different concentrations (45, 40, 35, 30, 25, 20, 15, and 10 mg/ml) of test plant extract (to form 5 x 105 bacteria per ml per tube) in duplicate, and for each concentration, negative control tube (C_od_) was made having 900 µl of BHI broth with 100 µl plant extract along with a positive control tube (B_od_) having 500 µl of BHI broth with 500 µl of bacterial culture containing 1 x 106 CFU/ml bacteria. These tubes were additionally incubated for one day at a temperature of 37°C. After that O.D. was taken at 600 nm of each tube followed by MIC calculation. The dose of extract showing inhibition around 99% was selected as MIC [12].

Statistical analysis

All statistical analyses along with table preparation were done in Microsoft Excel 2016 for Windows (Microsoft Corp., Redmond, WA).

## Results

**Figure 1:**
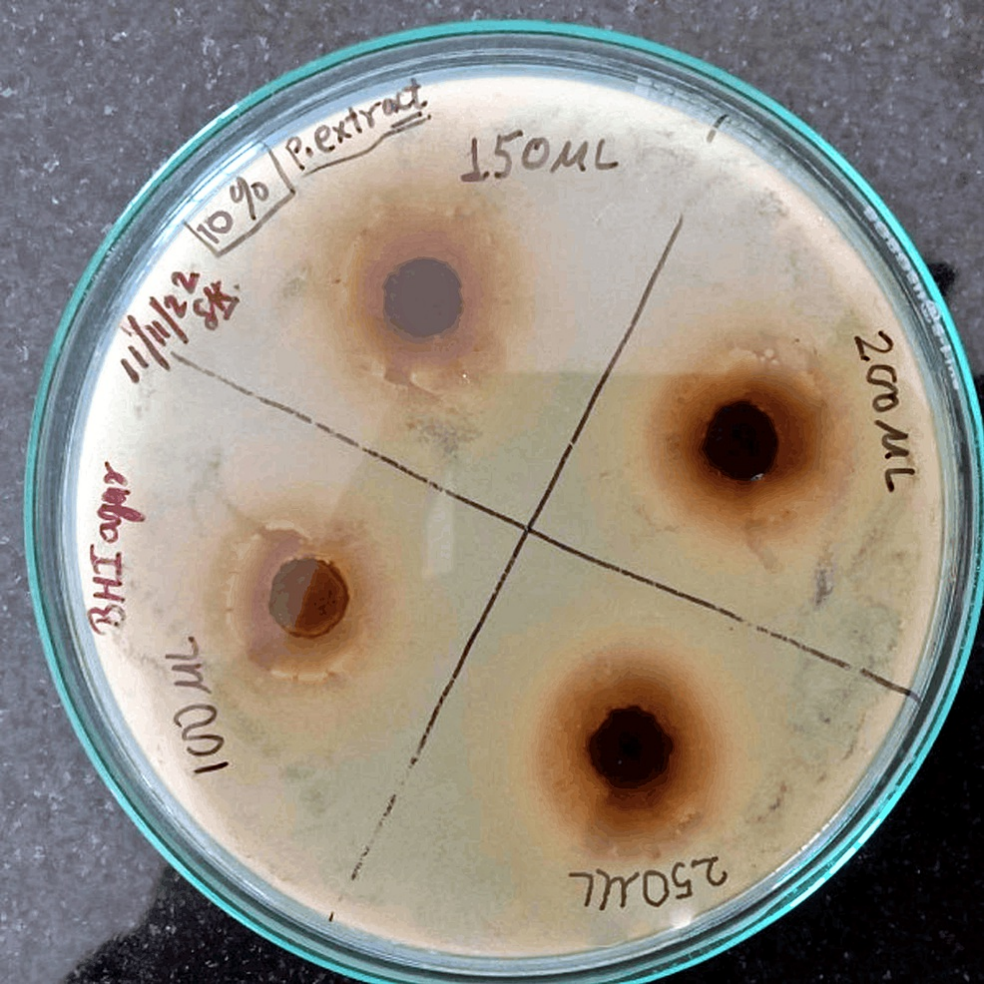
The zones of inhibition measured in millimeters (mm) for Madhuca longifolia extract.

The zones of* *inhibition for *Madhuca longifolia* extract at different concentrations are shown in Figure 1. The antibacterial activity was studied in two ways: (i) by measuring the zone of inhibition and (ii) by determining the MIC value. The diameter of zones of inhibition was measured in millimeters using a measuring guide in agar plates.

**Figure 2:**
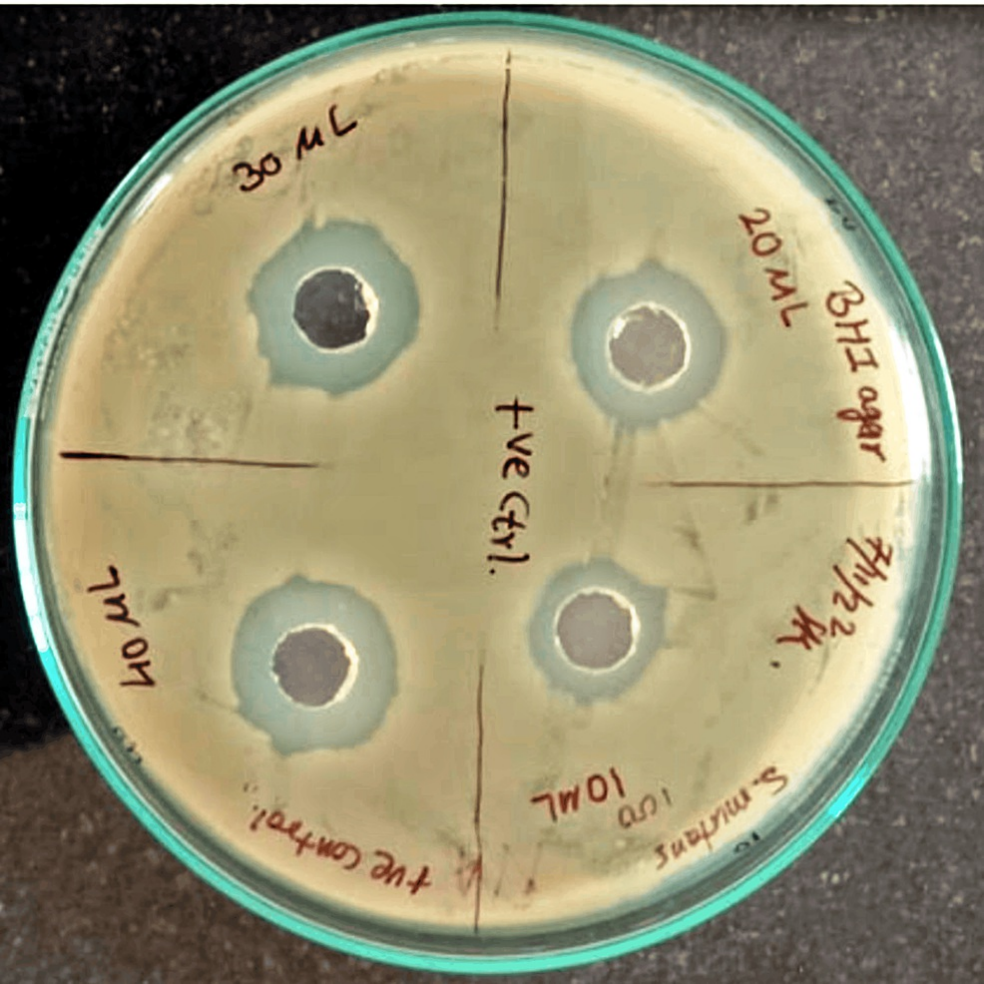
The zones of inhibition measured in millimeters (mm) for 0.2% chlorhexidine.

The zones of inhibition for 0.2% chlorhexidine are shown in Figure 2. The zone of inhibition formed is measured in millimeters, as shown on the agar plate.

**Figure 3:**
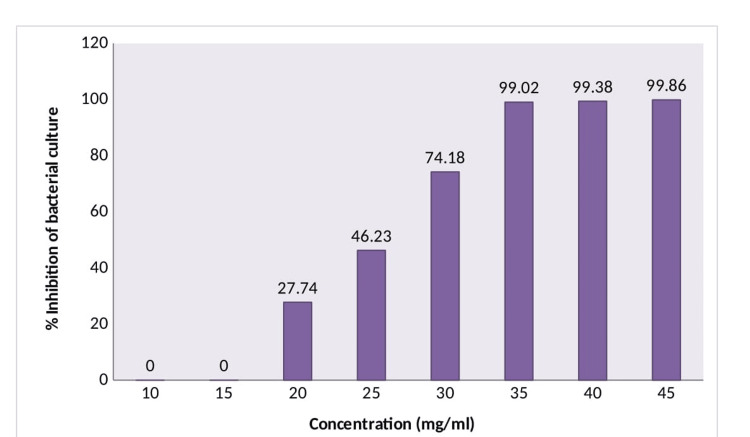
MIC of Madhuca longifolia by the macrodilution method. MIC: minimum inhibitory concentration.

The zones of inhibition for *Madhuca longifolia* stem twig extracts and chlorhexidine at 0.2% concentration at different concentrations and different volumes and MIC of *Madhuca longifolia* are shown in Tables 1-3, respectively. The minimum zone of inhibition of 11 mm was seen at 2.5% concentration and 100 µl volume of extract and the maximum zone of inhibition of 20 mm was seen at 10% concentration and 250 µl volumes. While for chlorhexidine at 0.2% concentration, the zone of inhibition obtained was 9.5 mm at 40 µl volume. The MIC of *Madhuca longifolia* by the macrodilution method is shown in Figure 3. The MIC value was found to be 35 mg/l.

**Table 1:** Zone of inhibition for Madhuca longifolia stem twigs extract.

Concentration (%)	Volume (µl)			
	100, zone of inhibition in mm, mean ± SD	150, zone of inhibition in mm, mean ± SD	200, zone of inhibition in mm, mean ± SD	250, zone of inhibition in mm, mean ± SD
2.5	11 ± 0.28	12 ± 0.42	12 ± 0.28	13 ± 1.70
5.0	11 ± 0.14	14 ± 0.28	16 ± 0.99	17 ± 1.13
7.5	12 ± 0.12	14 ± 0.57	17 ± 0.14	18 ± 0.14
10.0	13 ± 1.56	14 ± 0.14	19 ± 0.57	20 ± 0.85

**Table 2:** Zone of inhibition for chlorhexidine at 0.2% concentration.

Volume (µl)	Zone of inhibition (mm)
10	6.5
20	7.5
30	8.5
40	9.5

**Table 3:** MIC of Madhuca longifolia by the macrodilution method. MIC: minimum inhibitory concentration.

S. No.	Concentration used (mg/ml)	Dry weight (mg)	Test (OD) average (T_od_)	Control (OD) average (C_od_)	Differences (OD) (D_od_) = (T_od_ – C_od_)	% inhibition = (B_od_ – D_od_)/B_od_ × 100
1	45	4.5	0.8998	0.8996	0.0002	99.86
2	40	4.0	0.7704	0.7695	0.0009	99.38
3	35	3.5	0.6789	0.6775	0.0014	99.04
4	30	3.0	0.5759	0.5380	0.0379	74.22
5	25	2.5	0.6815	0.6025	0.079	46.26
6	20	2.0	0.5867	0.4805	0.1062	27.76
7	15	1.5	0.5370	0.3900	0.1470	0
8	10	1.0	0.4905	0.3435	0.1470	0
Bacterial culture (B_od_)			0.147			

## Discussion

The paradigm is being shifted to natural plant derivatives from synthetic antibiotics with significant side effects. Therefore, there is a plethora of reasons for the world to look back into our plant products and derivatives with negligible side effects and proven effectiveness. The chewing stick of some unique plants like neem is unequivocally used by masses of people across the globe. One such plant used in our area is *Madhuca longifolia,* whose stem twigs were examined to find out its antibacterial activity. The binary ways to assess antibacterial efficacy are as follows: by calculating the diameter of the zone of inhibition using the cup plate method and by determining the MIC value using the macrodilution procedure, which was followed in our study as per customary protocol [9,11].

Previous literature has mentioned the cup plate method as a standardized procedure to evaluate the efficacy of antibacterial products and was used in the present study [13-15]. However, Balouiri et al. have criticized it and found it inappropriate to establish MIC and also mentioned the impossibility of quantifying the total of the antimicrobial getting diffused into agar medium [9]. In this work, the control chosen was chlorhexidine gluconate at 0.2% concentration, which revealed a zone of inhibition of 9.5 mm at 40 µl volume. Simultaneously, with 10% concentration of *M. longifolia*, the zone of inhibition was 20 mm at 250 µl volume. In our study, the macrodilution method was used. The value recorded is 35 mg/ml. The *Madhuca longifolia* plant extract showed a noticeable antibacterial bustle and can be thought of as a toothbrush substitute in upcoming future.

Kendre et al. and Dalvi et al. have published review articles on the phytochemical study of *Madhuca longifolia* and reported that the plant extract is enriched with vital organic compounds like vitamins, sugar, protein, steroids, and other necessary growth factors, such as terpenoids, saponins, and phenolic compounds, because of which it has properties like anti-inflammatory, analgesic, antioxidant, anti-hyperglycemic, hepatoprotective, spasmolytic, anticonvulsant, and anticancer activities. In addendum to this, mahua flowers are extensively used in the making of liquor and varied foodstuffs in the food business [7,16]. Jyothi et al. extracted saponins from *M. longifolia* and reported antimicrobial properties against *Streptococcus mutans*, *Streptococcus salivarius*, *Staphylococcus aureus*, and *Lactobacillus acidophilus* [17]. Sikarwar et al. have prepared a list of 49 plants used as toothbrushes in India, which included *M. longifolia* twig as one with the best taste and useful as a toothbrush [5]. Devi et al. reported that the bark of this plant contains essential triterpenoids, alcohol, and ester derivatives, because of which this plant extract is rich in anti-inflammatory properties [18]. In our study, an aqueous extract of *M. longifolia* was used. The minimum zone of inhibition achieved was 11 mm, which as per the previous study is an effective antibacterial agent [9,19].

Moreover, there are some limitations in the study. The most inevitable one is that it is an in vitro study and dental caries has a multifarious etiology with varied interactions and mechanisms involved, hence the results cannot be blind fondly extrapolated on the population. Secondly, this is the first study done on *M. longifolia* evaluating its antibacterial potential against *S. mutans*, so we advise more advanced clinical studies on this plant.

## Conclusions

The present investigation shows beyond doubt that *Madhuca longifolia* possesses ample antibacterial worth against *Streptococcus mutans*. As the *Madhuca longifolia* plant is ecologically safe, cost-effective, and healthy, it can be of great benefit to oral hygiene and health.
